# Overcoming the Blood-Brain Barrier: Functionalised Chitosan Nanocarriers

**DOI:** 10.3390/pharmaceutics12111013

**Published:** 2020-10-23

**Authors:** Anna E. Caprifico, Peter J. S. Foot, Elena Polycarpou, Gianpiero Calabrese

**Affiliations:** School of Life Sciences, Pharmacy and Chemistry, Kingston University London, Penrhyn Road, Kingston upon Thames KT1 2EE, UK; A.Caprifico@kingston.ac.uk (A.E.C.); P.J.Foot@kingston.ac.uk (P.J.S.F.); E.Polycarpou@kingston.ac.uk (E.P.)

**Keywords:** chitosan nanocarriers, chitosan functionalisation, blood-brain barrier, receptor mediated transport, adsorptive mediated transcytosis

## Abstract

The major impediment to the delivery of therapeutics to the brain is the presence of the blood-brain barrier (BBB). The BBB allows for the entrance of essential nutrients while excluding harmful substances, including most therapeutic agents; hence, brain disorders, especially tumours, are very difficult to treat. Chitosan is a well-researched polymer that offers advantageous biological and chemical properties, such as mucoadhesion and the ease of functionalisation. Chitosan-based nanocarriers (CsNCs) establish ionic interactions with the endothelial cells, facilitating the crossing of drugs through the BBB by adsorptive mediated transcytosis. This process is further enhanced by modifications of the structure of chitosan, owing to the presence of reactive amino and hydroxyl groups. Finally, by permanently binding ligands or molecules, such as antibodies or lipids, CsNCs have showed a boosted passage through the BBB, in both in vivo and in vitro studies which will be discussed in this review.

## 1. Introduction

Neurological disorders, such as stroke, dementia, and tumours, are the main cause of disability and death worldwide, as found by the Global Burden of Diseases, Injuries, and Risk Factors Study (GBD) 2019 [1]. Currently, these diseases affect more than one billion people worldwide and no effective therapies are available [2]. Indeed, most of the therapeutic agents that are usually employed to treat neurological disorders show severe side-effects, including diabetes, seizures and toxicity, along with inability to reach the brain due to the presence of the blood-brain barrier (BBB) [3]. However, the BBB allows for passive diffusion via transcellular transport only to lipophilic molecules with molecular weight (MW) up to 600 Da and paracellular transport to hydrophilic molecules with MW lower than 200 Da [4]. In addition, in order to cross the endothelium, the molecule needs to contain fewer than five hydrogen bond donors and less than ten hydrogen bond acceptors. Finally, the calculated lipophilicity should be less than five [5].

Therefore, research on the potential therapeutic benefit of small agents is gradually emerging, especially in the context of modulation of the cellular redox state, involved in the initiation of several neurodegenerative disorders, as extensively discussed by Schiavone and Trabace, 2018 [6]. Moreover, a cocktail of small molecules, including forskolin, has also been investigated for its ability to reprogram glioblastoma cells into neurons [7]. Wang et al., [8] developed an in silico method to predict the permeability of small molecules towards the BBB using unbiased atomic molecular dynamics simulations. The data were compared to a standard in vitro BBB model while using a transwell assay, which indicated that this new in silico tool can efficiently predict the in vitro permeability assessment of small molecules for drug development [8].

However, only 2% of small molecules can cross the BBB, such as opiates, anxiolytics, and antipsychotics [9]. Almost 100% of macromolecules including antibiotics and anti-tumoral are unable to reach the brain [9,10]. Only 5% of over 7000 drugs that are contained in the Comprehensive Medicinal Chemistry (CMC) database can treat neurological disorders, primarily depression, schizophrenia and insomnia [10]. Several promising drug candidates that have shown significant pharmacological activity in experimental investigations fail in clinical trials due to insufficient transport through the BBB [11].

In fact, the BBB is provided with efflux pumps (a class of ATP-binding cassettes (ABC), such as P-glycoproteins-P-gp), which expel harmful substances and numerous therapeutic actives to the exterior of the brain, representing the main obstacle for drug delivery to the brain [12].

Novel technologies are needed to ensure efficient drug delivery, optimal distribution, and concentration of drugs into the brain because of the complexity of the BBB and the challenges it imposes on the treatment of disorders of the Central Nervous System (CNS) [13]. Several approaches were developed to mechanically bypass the BBB; these include transcranial catheter implantation and osmotic and biochemical disruption of the BBB. However, these strategies can be deleterious to the brain, leading to chronic neuropathological changes or seizures [11]. The intranasal route for delivering therapeutics to the brain has attracted considerable attention in the literature; however, animal studies have led to ambiguous findings [11]. For instance, the conditions of delivery applied to animals (e.g., high pressure applied or obstructing the oesophagus to preclude swallowing) were unsuitable for humans [11].

**Figure 1:**
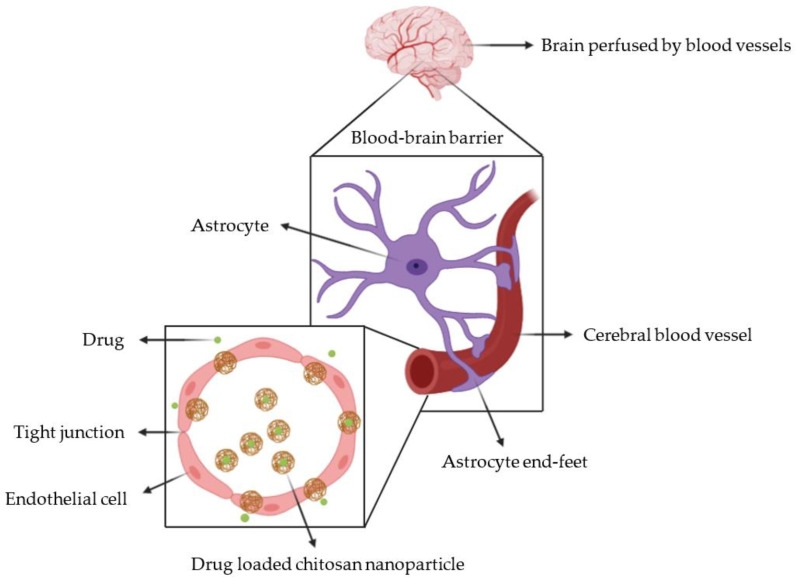
The cross-section of the cerebral blood vessel shows the interaction of drug loaded chitosan nanoparticles with the blood-brain barrier.

Hence, the need to develop drug delivery systems, such as nanoparticles (NP), micelles, or in situ gel, which can overcome drugs’ limitations [3]. Several types of nanocarriers (NC), having a size between 100 and 1000 nm, have been developed to deliver therapeutics [14]. NPs are able to cross the BBB and specifically target the brain due to their nanoscale dimensions, hence limiting therapeutics’ side-effects [12,15]. A phenomenon known as the “enhanced permeability and retention” (EPR) effect of the disrupted BBB is responsible for the passive accumulation of NCs into the disorganized architecture of brain tumours [16]. NCs can alter the drug’s pharmacokinetic profile by avoiding efflux pumps [17]. Indeed, the mechanisms of crossing only depend on the physicochemical and biomimetic characteristics of the NC, since the drug is hidden by it (Figure 1) [9].

A promising non-invasive method is to modify the surface of NCs with specific targeting molecules, such as peptides or antibodies, which target a specific receptor on the BBB, promoting drug internalization [12,18]. Considerable attention has been paid to colloidal species as potential brain drug delivery systems due to their ability to control drug release and specifically target the brain [18].

Polymeric NCs that are based on low MW (LMW) chitosan (Cs) are promising candidates for the non-invasive transport of therapeutics through the BBB, due to their numerous advantages, including low toxicity, biodegradability, biocompatibility, and mucoadhesivity [19]. A recent review described chitosan-decorated drug delivery systems as effective in delivering drugs to the brain, owing to chitosan’s mucoadhesive property [20]. However, chitosan offers several advantages besides mucoadhesion, including the ease of functionalisation owing to functional groups on its structure, which further improve chitosan-based nanocarriers’ (CsNC) properties. A review on this topic has not yet appeared in the literature. Therefore, this review reports on the most recent strategies for improving the permeability and selective transport of therapeutic agents across the BBB, while using NCs based on chitosan functionalised with specific targeting molecules.

## 2. The Blood-Brain Barrier

The CNS is entirely protected by the BBB. This is a unique microvasculature composed of highly specialised endothelial cells that are held closely together by continuous tight junctions (TJ), such as zonula occludens, claudins, and adherens junctions (AJ). TJs contribute to the tightness of the BBB since ensuring paracellular permeability by establishing a high electrical transmembrane resistance (TEER), being >1500 Ωcm^2^, the highest value when compared to other endothelial barriers [9,21]. The tightness of the BBB is also provided by other cell types including pericytes, microglial cells, astrocytes and neurons with which endothelial cells communicate, forming the neurovascular unit [22]. The BBB prevents harmful substances to reach the brain, maintaining the homeostasis of the CNS and ensuring that the brain is supported with nutrients. Indeed, endothelial cells express transporters for nutrients (e.g., glucose and amino acids) and receptors for larger molecules (e.g., transferrin receptor (Tf-R) for iron uptake) on their surface. Because the brain is in high demand for nutrients, the BBB overexpresses receptors and transporters to ensure that nutrients are delivered to the brain at an acceptable concentration [12]. These transport routes have been explored for the transport of pseudo-nutrients through the BBB. For instance, the prodrug l-DOPA (l-3,4-dihydroxyphenylalanine) targets the l-amino acid transporter and it is used to treat Parkinson’s Disease [23].

## 3. Chitosan Nanocarriers

**Figure 2:**
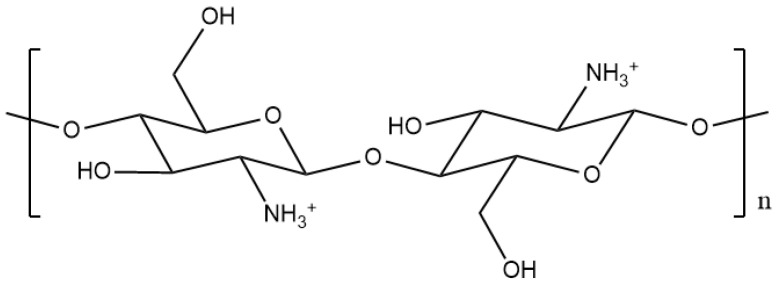
Structure of pristine chitosan.

Chitosan is derived from partial *N*-deacetylation of chitin in hot alkaline media, resulting in a copolymer that consists of *N*-acetyl-glucosamine and *N*-glucosamine units linked by *β*-(1′4)-glycosidic bonds. Figure 2 shows its structure. The reaction conditions for its production determine the degree of deacetylation (DDA) and MW of chitosan [24]. The commercially available chitosan has a DDA that ranges between 70–85%. It is soluble in dilute acidic solutions, but insoluble at physiological pH. The polycationic amino groups confer upon chitosan the property of mucoadhesion, namely the establishment of ionic interactions with anions on the cell surface or mucous membrane, which increase the residence time at the target site, thereby enhancing membrane absorption [25].

The protonated amino groups are also involved in the formation of NPs by ionic gelation. This is a widely used technique, since very mild conditions are required. It relies on electrostatic interactions between the positive charges on chitosan and the negative charges on a non-toxic cross-linker, such as sodium tripolyphosphate (TPP) [26]. When using this technique, the determination of the ratio Cs/TPP and pH of the solution is key for optimizing NPs’ physicochemical properties, such as size and zeta potential (ZP) [27]. Indeed, the size and ZP play a key role in the endocytosis of NPs by brain endothelial cells since it was found that, to be effective in crossing the BBB, NPs should have a size smaller than 200 nm and a positive ZP value [28,29]. For instance, Jahromi et al. [30] employed ionic gelation to generate CsNPs of suitable size and ZP for the transport of methotrexate (Mtx) through the BBB. Mtx is unable to cross the BBB, being a substrate of several efflux transporters on the BBB, especially the P-gp [31]. The resulting NPs had a size of 134.1 nm and ZP of +22.8 ± 6.55 mV, with both values being within the range required for BBB crossing and avoiding clearance from the reticuloendothelial system (RES) [32]. Mtx-loaded CsNPs were then injected into rats which were also treated with verapamil, a P-gp inhibitor. Plasma and brain were then isolated and the concentration of Mtx was measured. It was found that in absence of verapamil the concentration of Mtx-loaded in CsNPs significantly decreased in the plasma and increased in the brain. when compared to free Mtx. This effect was even more pronounced upon treatment with verapamil and was time-dependent. The authors [30] suggested that over a short incubation time and in the absence of verapamil, Mtx-loaded CsNPs released their cargo next to the BBB at a high concentration that saturated the P-gp activity, increasing the amount of Mtx that was delivered to the brain within that time.

## 4. Functionalization of Chitosan

Chitosan’s functional groups (amino, primary, and secondary hydroxyl groups) allow a wide range of chemical modifications such as acylation, tosylation or *O*-carboxymethylation as well as functionalization with antibodies, ethers, or lipids, to be performed on its structure [18,33]. Indeed, besides the size and ZP, CsNPs are usually modified with other materials, so they can selectively transport drugs across the BBB. This is needed, because the most frequent method to administer CsNCs for brain targeting is by intravenous (i.v.) injection, owing to the large number of capillary vessels that perfuse the brain [21]. Once in the blood, cell toxicity depends on the concentration and the MW of CsNCs. Therefore, CsNCs can be highly unstable in the systemic circulation, due to activation of the RES. To overcome this issue, the surface of NPs is frequently modified by using polyethylene glycol (PEG), a hydrophilic polyether compound, whose presence does not damage brain cells, in both in vitro and in vivo studies [34,35]. The employment of PEG can reduce the cytotoxicity as well as macrophage uptake by preventing the interaction of NCs with cellular or serum proteins [36]. Finally, because the poor solubility and permeability of chitosan in physiological pH limits its clinical use, functionalization of the chitosan structure helps to decrease systemic side-effects while enhancing the drug loading and cellular uptake along with controlling drug release [18].

### 4.1. Trimethylated Chitosan

**Figure 3:**
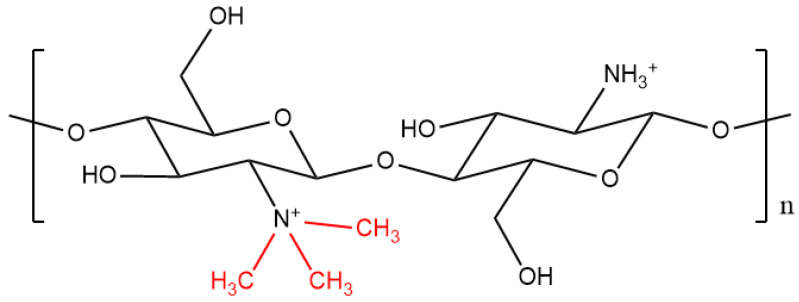
Structure of *N*,*N*,*N*-trimethyl chitosan (methyl groups are shown in red).

*N*,*N*,*N*-trimethyl chitosan (*N*-TMCs) is a derivative of chitosan, which is produced through amino functionalisation with methyl iodide, at high temperature under alkaline conditions. However, the standard reaction for modification of chitosan to obtain *N*-TMCs makes use of toxic solvents, such as *N*-methyl pyrrolidinone. This reaction produces *N*-TMCs whose degree of quaternization (DQ) can be increased by having a greater number of reaction steps or a longer reaction time, or by using batches with different DDA. Figure 3 shows the structure of *N*-TMCs.

When increasing the DQ, *O*-methylation on the hydroxyl groups can occur, leading to a less soluble compound. However, even a *N*-TMCs with a DQ of 10% showed numerous advantages that were attributed to its permanent positive charges. These advantages include improved solubility in a wide range of pH, porosity, strength, absorption efficiency, and mucoadhesivity [37]. Moreover, a low degree of quaternization can increase the loading efficiency of drugs [38]. The permanent positive charges can facilitate absorptive mediated transcytosis (AMT) of NPs and, because of this, *N*-TMCs can be used to increase the transport of actives to the brain [29]. However, higher positive charges can be obtained by increasing the degree of quaternization of chitosan, which, on the other side, induce a larger particle diameter [38]. Therefore, reaction conditions for the preparation of *N*-TMCs must be finely controlled, so as to obtain a modified polymer that leads to the formulation of NPs with the wanted physical properties and drug loading efficiency. Wang et al. [38] employed *N*-TMCs to formulate NPs loaded with the anti-neuroexcitation peptide (ANEP) for the treatment of epilepsy. ANEP or TMCs were labelled with fluorescein isothiocyanate (FITC) to allow in vivo tracking of the NPs in mice. Following i.v. injection of fluorescent free ANEP (control) and ANEP loaded in NPs, the mice were sacrificed and the brain, along with the major organs, were isolated. Stronger fluorescence was recorded in the brain of mice treated with ANEP loaded in NPs than ANEP free in solution. This suggested that free ANEP is not able to cross the BBB whereas, when loaded in TMCs NPs, the strong positive charge (+31 mV) allowed AMT to occur, successfully delivering ANEP to the brain [38].

Other studies make use of *N*-TMCs as a coating on the surface of NPs for BBB crossing. For instance, *N*-TMCs was used to modify the surface of poly(d,l-lactide-*co*-glycolide) (PLGA), a biodegradable polymer [29]. One of the disadvantages of the conjugation reaction of TMCs with PLGA is the use of 1-(3-dimethylaminopropyl]-3-ethyl carbodiimide hydrochloride (EDAC), which is hazardous. Moreover, highly reactive intermediates are produced following the reaction of EDAC with the carboxylic groups on PLGA to form an amide bond with chitosan. After coating with *N*-TMCs, the NPs had a size of 150 nm and ZP of about +20 mV. Uncoated PLGA NPs were taken as control, being characterized by a negative ZP (−20 mV). In vivo studies were performed and, to assess their effectiveness to reach the brain, NPs were labelled with 6-coumarin and then i.v. injected into mice. The images of brain sections showed that the coating with *N*-TMCs caused a greater accumulation of PLGA NPs in the brain than uncoated PLGA NPs. Strong fluorescence was recorded upon treatment with *N*-TMCs modified PLGA NPs, which suggests that the positive charge of *N*-TMCs on PLGA NPs played a key role in enhancing NPs’ uptake by cerebral endothelial cells [29]. Moreover, the *N*-TMCs offered a hydrophilic surface that increased the blood retention time, enhancing the accumulation of NPs in the brain [29,39].

Ramalingam et al. [40] employed *N*-TMCs to coat the surface of solid lipid NPs. The successful coating with *N*-TMCs was confirmed by an increased size of NPs (412 nm) when compared to uncoated NPs (138 nm). This new drug delivery system was designed for the oral administration of curcumin aimed to reach the brain, for the treatment of Alzheimer’s disease and gliomas. NPs were loaded with curcumin, and their biodistribution after oral administration was assessed by in vivo studies while using mice. After oral administration of NPs, the mice were sacrificed, and their plasma and their brains were isolated to measure curcumin concentration. The results showed that the bioavailability of curcumin increased upon loading in *N*-TMCs coated solid lipid NPs, meaning that the coating could protect curcumin from gastrointestinal degradation. As a result, the concentration of curcumin in the brain was higher than that obtained using the free drug or loaded uncoated NPs. These results suggest that *N*-TMCs coated solid lipid NPs were able to enhance the oral bioavailability and brain accumulation of curcumin, by-passing the BBB [40]. However, the four-fold increase in size that was caused by coating with *N*-TMCs can impede the passage of NPs through the BBB, since to efficiently cross the BBB, NPs’ size should be lower than 200 nm [28,29].

Most recently, *N*-TMCs was used to functionalise the surface of retinoic acid (RA)-loaded solid lipid NPs for treatment of glioblastoma multiforme [41]. RA is a potential chemotherapeutic agent which binds to its receptor on cancer cells, inhibiting their growth [42]. Since its main limitation is its solubility in water, RA was encapsulated in lipid NPs that were known to cross the BBB due to their small size (100–300 nm) [43,44]. After coating with *N*-TMCs, the NPs’ size increased from 148 to 214 nm and the ZP increased from −21 mV to +20 mV, confirming that the coating with *N*-TMCs was successful. Cell cytotoxicity and apoptosis studies were performed on human brain glioblastoma cells: greater cytotoxicity was observed upon treatment with *N*-TMCs coated NPs, owing to their positive charges that improved RA uptake by cancer cells. However, there was no study of the uptake of *N*-TMCs coated NPs in an in vitro model of BBB, or an in vivo investigation to show the crossing of NPs through the BBB. The existence of this crossing can be inferred from the in vivo study carried out by Ramalingam et al. [40]. However, the size of *N*-TMCs coated NPs in Liu et al. [41] study was half that of Ramalingam et al. [40], which suggests a boosted passage of NPs through the BBB. The difference in size between the two studies may be attributed to the higher concentration of *N*-TMCs that were added in the solution of NPs by Ramalingam et al. [40] (50:1, *w*/*w*) compared to Liu et al. [41] (30/1, *w*/*w*). One can deduce that the concentration of *N*-TMCs added during the coating process of lipid NPs plays a key role in determining the size of NPs and, hence, their ability to be successfully employed in the delivery of therapeutic agents to the brain.

### 4.2. Alkylglyceryl-Modified Chitosan

Alkylglycerols (AGs) are glycidyl ether lipids occurring naturally in shark or dogfish liver oils, in human or cow milk, and hematopoietic organs, including liver, spleen, and bone marrow [45]. Their structure is characterized by a fatty acid, whose chain length and number of double bonds can vary, linked to the α-position of a glycerol backbone by an ether bond [46]. AGs have several biological effects in patients with cancers. For instance, the administration of AGs alongside radiotherapy to treat uterine cervical cancer resulted in reduced toxicity given by radiotherapy and increased survival time [47]. Other biological effects involve the stimulation of the immune system by increased production of blood leukocyte and thrombocytes, activation of macrophages, and anti-tumour response [46]. Concerning the BBB, it has been found that the treatment with AGs led to better transport of therapeutics to the brain, probably due to the modulation of TJs caused by their hydrophobic interaction with the lipid cell bilayer of endothelial cells [48,49].

**Figure 4:**
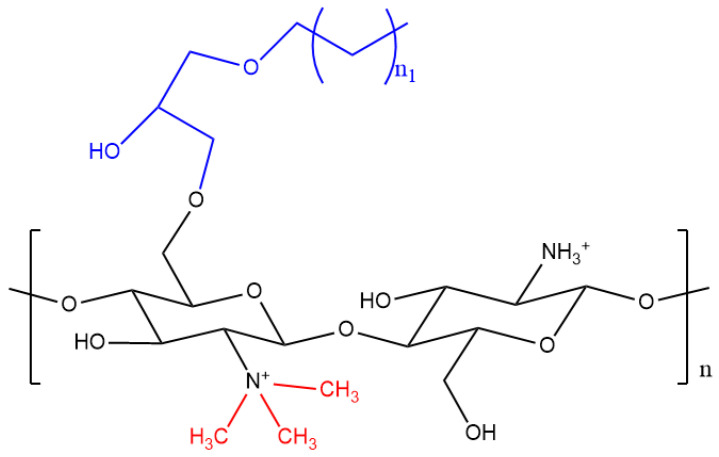
Structure of *N*,*N*,*N* trimethyl chitosan (methyl groups are shown in red) functionalised with an alkylglycerol (shown in blue) where “n_1_” represents either four, five, or eight alkyl chain.

Molnar et al. [50] and Lien et al. [51] created NPs that combined the properties of both LMW chitosan and AGs for an increased permeability of therapeutics across the BBB. Chitosan (75–85% DDA) was functionalised with a specific chain length given by butyl, pentyl and octyl glycidyl ethers [50,51]. This was done through *O*-regioselective grafting with the primary hydroxyl groups of chitosan and protection of the amino groups by phthaloylation. Positive charges on the AGs-Cs were also introduced to generate *N*-TMCs with different DQ, avoiding *O*-methylation. Figure 4 shows the structure of alkylglyceryl-modified trimethyl chitosan (AG-TMCs).

*N*-TMCs were used to study the effect of ionisation on the stability of NPs and their solubility in water. The novel polymers were employed to form NPs by ionic gelation at a polymer/TPP ratio of 6/1, resulting in NPs size range of 200–300 nm. The ZP of AGs-CsNPs was lower (+37 to +41 mV) than CsNPs (+51 mV). Regarding NPs that were formulated with the AGs-TMCs compounds, a polymer with DQ greater than 44% showed higher solubility in water but it was impossible to separate the NPs by centrifugation. NPs were only obtained with a DQ lower than 35%, with increased stability over a wide range of pH (4–8) in terms of size and ZP. An in vitro model BBB, based on mouse brain microvascular cells, was employed in order to study the passage of NPs (AGs-CsNPs and AGs-TMCsNPs, CsNPs were used as control). To track the intracellular route that was undertaken by NPs, Evans Blue (EB), a fluorescent anionic dye unable to cross the BBB, [52] was encapsulated in the NPs during the ionic gelation process. The results showed that EB loaded AGs-CsNPs were unstable in the cellular culture medium, since some aggregation occurred, which was possibly caused by increased lipophilicity from the AG chain, leading AG chains to attract one another. In contrast, EB-loaded AG-TMCsNPs proved to be more stable than AGs-CsNPs, since the size and the ZP were retained at physiological pH. Indeed, owing to the permanent positive charges introduced by *N*-TMCs, NPs repelled each other. Confocal images showed that the EB signal (blue) that was associated with the presence of NPs was detected in the perinuclear region and in the Golgi apparatus, indicating that both AGs-CsNPs and AGs-TMCsNPs were successfully engulfed by endothelial cells. Finally, upon staining the TJs, their rearrangement appeared to be more pronounced when endothelial cells were treated with AGs-NPs than CsNPs, suggesting boosted permeability of AGs-CsNPs and AGs-TMCsNPs through the BBB model than pristine CsNPs [50,51]. However, a drawback of both studies is the use of murine endothelial cells that do not reflect the same characteristics as human endothelial cells in terms of TEER and expression of efflux pumps. Therefore, further studies need to be performed on human endothelial cell lines, such as the hCMEC/D3 monolayer [53] or in vivo investigations, in order to establish the clinical relevance of AGs-TMCs NPs. Moreover, a suitable drug should be incorporated into the AGs-TMCs NPs, rather than a fluorescent dye, such as EB. The presence of lipophilic chains on the chitosan backbone could accommodate drugs with poor water solubility, such as RA or *O*^6^-benzylguanine, which are in the pipeline for the treatment of brain tumours [42,54].

## 5. Coated Chitosan Nanoparticles for Receptor Mediated Transport (RMT)

### 5.1. Antibody-Coating

**Figure 5:**
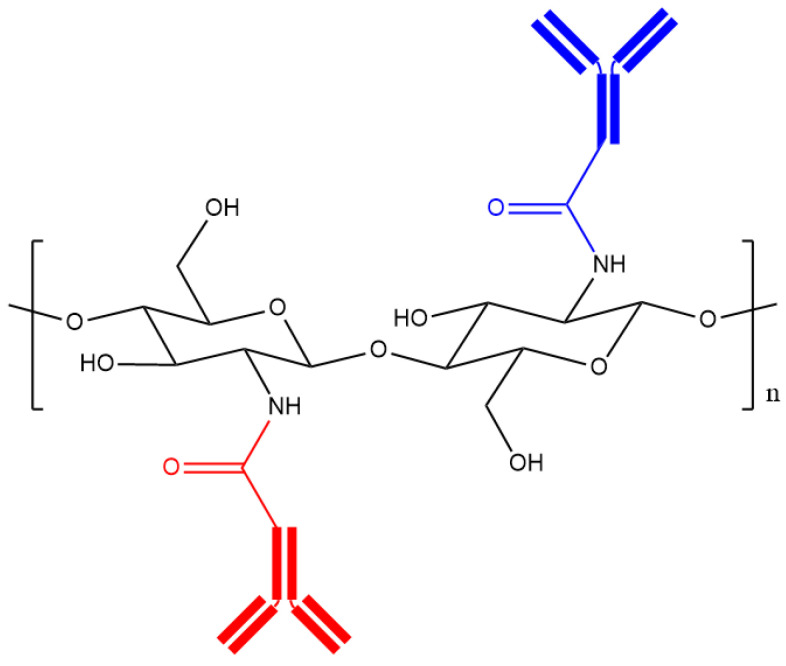
The structure of chitosan functionalised with two different antibodies (an antibody is shown in red and the different antibody is shown in blue).

The efficiency of drug-loaded NPs in crossing the BBB has been improved by engineering the surface of NPs with targeting ligands (Figure 5) [55].

Agyre et al. [56] coated the surface of CsNPs with a biosensor detecting amyloid deposits, located on the cerebral vasculature and brain parenchyma. Amyloid deposits are responsible for Alzheimer’s disease and cerebral amyloid angiopathy [57,58]. The F(ab’)2 fragment of Aβ Ab (IgG4.1) modified with putrescine, referred to as pF(ab’)24.1 (pF), was employed to coat NPs, since pF can cross the BBB and bind to the amyloid deposits [59]. An in vitro model of BBB was generated using bovine brain microvascular endothelial cells, grown on Transwell inserts. The cellular uptake of NPs was assessed by loading FITC labelled bovine serum albumin (BSA) into both pF-CsNPs and CsNPs (control). The flow cytometry results showed that the intracellular fluorescence was significantly higher after treatment with FITC-BSA-pF-CsNPs than the control, suggesting higher cellular uptake of FITC-BSA-pF-CsNPs than FITC-BSA-CsNPs. Permeability studies that were conducted by Z-stack images confirmed that the control was unable to permeate the cell monolayer, while the pF-CsNPs were successfully internalized by endothelial cells. Moreover, the radioisotope ^125^I was used to label NPs that were intravenously administered to mice and it was shown that pF-CsNPs were able to accumulate within the brain, since higher radioactivity was recorded in the brains of mice treated with pF-CsNPs than in those that were treated with the control (CsNPs) [56]. In this work, the coating of CsNPs with the biosensor was undertaken at pH 6.5, in order to reduce the density of positive charges on chitosan and increase the electrostatic interactions with the cationic antibody. The antibody was not conjugated with the chitosan polymer by a chemical reaction, meaning that the dissociation of the antibody from the NP might be affected by the variation of pH of the medium in which the coated NPs are dispersed. For instance, upon internalization of the NPs by the endothelial cells, NPs may undertake the lysosomal pathway wherein the pH is below 6, triggering dissociation of the antibody from the surface of the CsNP. As a result, the targeting efficiency of CsNPs may be lost.

Another widely used targeting ligand for the brain is the anti-Tf-R antibody (Ab), which is highly selective towards the Tf-R, located on cerebral endothelial cells and glioblastomas [35]. For instance, Yemisci et al. [60] employed CsNPs modified with an anti-Tf-R Ab and PEG to deliver an irreversible inhibitor peptide (N-benzyloxycarbonyl-Asp(OMe)-Glu(OMe)-Val-Asp(OMe)-fluoromethyl ketone (Z-DEVD-FMK)) to the brain [61]. This inhibitor cannot cross the BBB; hence, it cannot reach the brain parenchyma in useful concentration [62]. It inhibits caspase-3 which mediates apoptosis and plays a key role in cerebral ischemia [62,63]. The ability of Z-DEVD-FMK loaded, anti-Tf-R Ab modified CsNPs to reach the brain was evaluated in vivo. The brain uptake was expressed as the fluorescence intensity given by NPs loaded with Nile Red (which emits fluorescence and it is easily loaded into NPs) [64]. Fluorescent NPs were i.v. administered to mice and the concentration of NPs in the brain was assessed. Strong fluorescence was found in the brains of mice that were treated with Z-DEVD-FMK loaded, anti-Tf-R mAb modified CsNPs. The passage of the fluorescent NPs through the BBB was also assessed by staining the anti-Tf-R Ab using a FITC conjugated anti-anti-Tf-R Ab. The brain sections were analysed by fluorescence microscopy and the anti-Tf-R Ab was found within the brain parenchyma, in the external surface of vessels, confirming that anti-Tf-R Ab CsNPs could successfully cross the BBB [60]. Finally, electron microscopy confirmed these results, showing that NPs appeared as 200 nm sized, dense spherical particles, located in both the cerebral endothelium and the brain parenchyma [60]. Conversely to Agyre et al. [56], in the work that was performed by Yemisci et al. [60], the anti-Tf-R Ab was conjugated to the CsNPs by the formation of maleimide groups on the Ab, implying a reduced dissociation of the Ab from the NPs. However, the conjugation reaction made use of water insoluble compounds, such as m-maleimidobenzoyl-N-hydroxysuccinimide, and no purification step was reported. Finally, no cell viability test or antibody dosage was performed in vitro or in vivo, respectively, to verify the safety of the formulated NPs. However, PEGylation of the Ab-conjugated CsNPs may increase their half-life and decrease the cytotoxicity of the vehicle [36]. In view of these considerations, Monsalve et al. [35] also used NPs coated with PEG in order to increase the retention time of NPs in the blood [36]; OX26 monoclonal antibody (mAb), an anti-Tf-R Ab was used to functionalise chitosan and form NPs by ionic gelation. The reaction occurred between the primary amino group of chitosan and succinic anhydride, forming amide bonds. The resulting carboxylic acid groups were left free to anchor the Ab on the NPs’ surface. The reaction of conjugation with the Ab involved the use of an acetate form of chitosan to produce succinic anhydride-conjugated chitosan. This was followed by the employment of the NHS-EDC technology for obtaining the Ab conjugated NPs. The method adopted by Monsalve et al. [35] involves many steps for the production of Ab-conjugated NPs to allow chitosan to accommodate the structure of the Ab on its backbone. Functionalised NPs showed a mean size of 150 nm with a low polydispersity index and positive ZP (+20 mV). These data were not significantly different from those of Ab-free NPs, suggesting that the functionalisation did not affect the NPs’ stability. In vivo experiments were performed by the administration of OX-26 mAb/FITC labelled CsNPs and control (unmodified FITC labelled CsNPs) to mice. Each section of the brain (hippocampus, cortex, striatum, corpus callosum, and thalamus) was analysed while using confocal microscopy. The images showed a higher accumulation of OX-26 mAb/FITC labelled CsNPs in the hippocampus compared to other sections of the brain and to the control. This result suggested that the functionalisation of CsNPs with OX26 led to NPs with higher efficiency in crossing the BBB in vivo, and the mechanism of proposed transport was receptor-mediated transcytosis [35]. In addition, images of the microvessels showed a strong association of functionalised CsNPs with microvessels’ surfaces, possibly due to strong interaction with the Tf-R on the brain endothelium [35].

Sahin et al. [65] modified the surface of CsNPs while using an anti-Tf-R mAb and tested their uptake by the human endothelial hCMEC/D3 cells, with further investigations on the cellular uptake mechanisms undertaken by the NPs. CsNPs were prepared by ionic gelation, achieving a size/ZP of 274 nm/30 mV for CsNPs (control) or 284 nm/34 mV for anti-Tf-R mAb modified CsNPs. The conjugation reaction adopted by Sahin et al. [65] did not involve the use of any toxic solvent or chemical reactions, since the Ab was conjugated with streptavidin before mixing it with the suspension of CsNPs.

NPs were labelled with the fluorescent dye Dil in order to perform in vitro cellular uptake studies. A moderate degree of aggregation was recorded due to ionic interactions between the positively-charged chitosan and the components of the cell culture medium, such as amino acids, serum proteins, and salts [66]. This may also result in the aggregation of CsNPs in the blood upon contact with plasma proteins; hence a way to reduce the positive charge on chitosan had to be adopted before performing in vivo studies. To assess the impact of NPs aggregation on the cellular uptake, the incubation time of cells with NPs was prolonged up to 3 h. Fluorescence microscopy was used to determine the cellular uptake, which was higher for anti-Tf-R mAb-modified CsNPs than the control. Indeed, the control was found on the cellular surface, whereas the anti-Tf-R mAb modified CsNPs were in the perinuclear region of cells. This result suggested that the cellular uptake occurred before aggregation could start. The cellular uptake mechanisms were investigated by incubating cells with different endocytosis intracellular pathway inhibitors, such as amiloride (inhibiting the micropinocytosis), chloropromazine (inhibiting the clathrin-mediated endocytosis, CME) or Mβ-cyclodextrin (blocking the caveolae-mediated pathway). It was found that the cellular uptake of anti-Tf-R mAb-modified CsNPs was inhibited upon treatment with chloropromazine and amiloride. Hence, receptor-mediated endocytosis was probably the main mechanism involved in the cellular uptake of anti-Tf-R mAb modified CsNPs by hCMEC/D3 cells, while micropinocytosis could occur upon the aggregation of NPs [65].

Gu et al. [67] employed dual ligands modified CsNPs to deliver therapeutic genes, such as small interfering RNA (siRNA), which inhibit immunodeficiency virus (HIV) infection in astrocytes. Chitosan was simultaneously functionalised with two Abs, the anti-Tf-R Ab to target the cerebral endothelial cells and the human bradykinin B2 receptor (B2-R) Ab to target the astrocytes [68]. Gu et al. [67] adopted a more straightforward method than Monsalve et al. [35] to conjugate the Ab to CsNPs: the formation of succinic anhydride-conjugated chitosan was avoided, and the EDC/NHS method was directly applied to the CsNPs, in the presence of the Ab. This showed that the intermediate succinic anhydride-conjugated chitosan is not needed to obtain an Ab conjugated CsNPs. After functionalisation with Abs and incorporation of siRNA, NPs appeared to be spherical, uniform in size (mean of 200 nm), and with positive ZP (+22 mV). An in vitro model of brain/BBB, constituted by a co-culture of human astrocytes cells and hCMEC/D3 cells, was used to simultaneously evaluate the transport of NPs through the BBB and their subsequent uptake by astrocytes. The siRNA was labelled with a fluorochrome, Cy3, before loading it into the NPs, so that the cellular uptake of NPs was easily tracked by the fluorescence intensity. Flow cytometry analysis showed that the uptake of the dual-ligand modified CsNPs by both cellular types was significantly higher when compared to no Ab or single Ab-modified NPs, suggesting that the presence of both ligands could be an effective strategy for siRNA-targeted delivery to the brain [67].

### 5.2. Surfactant-Coating

Polymeric NPs that were coated with a surfactant, such as polysorbate 80 (P80), have extensively been used to deliver therapeutics efficiently to the brain while decreasing RES removal [69]. P80 is regarded as the gold standard surfactant for the delivery of NPs to the brain, since it increases the retention of NPs in the brain endothelium [14,70,71]. Indeed, following the incubation of P80-coated NPs with human citrate-stabilised plasma, Apo lipoproteins (ApoE and ApoB) contained in the plasma were adsorbed by P80-coated NPs [72]. These Apo lipoproteins, being on the surface of NPs, play a similar role as targeting ligands, since they are recognized by their own receptors on endothelial cells, allowing for NPs to be taken up by RMT [69]. Another proposed mechanism of action of P80 to increase the passage across the BBB is the inhibition of the efflux transporters such as P-glycoproteins [72].

An early investigation that tested P80 coated CsNPs passage through the BBB was done [73]. P80 coating of NPs (sized 80 nm) was performed after NPs generation simply by physical adherence of P80 on CsNPs surface. However, the extent of coating could not be well defined. This may have impact on the charge of the NPs that was not reported by Soni et al. NPs were labelled with ^99m^Technetium to perform biodistribution studies in mice. The radioactivity in each organ (brain, lungs, liver, spleen, and bone) was measured while using a gamma spectrometer. P80 uncoated NPs were taken as control and found to accumulate to a greater extent in the organs of the RES as compared to P80-coated NPs, suggesting that P80 coating decreased the removal of NPs. More importantly, the radioactivity in the brain associated with P80-coated NPs was 5-fold higher than uncoated NPs and increased with time, indicating that P80 coating enhanced the transport of CsNPs through the BBB [73].

Trapani et al. [16] loaded Mtx into P80-coated CsNPs for the treatment of brain tumours. In contrast to Soni et al. [73], a 20 times lower concentration of P80 was used in the study [16], in order to decrease the side effects of the surfactant. Moreover, the coating with P80 was performed on the chitosan polymer before forming NPs so that the particles had a mixture of polymer and surfactant on their surfaces, rather than pure surfactant. P80-coated CsNPs showed a lower ZP than uncoated NPs, confirming the presence of the surfactant on NPs. Therefore, coating with P80 did not completely mask the positive charge of CsNPs. The transport of NPs across the BBB was evaluated in Madin–Darby canine kidney cells, which are widely used for in vitro models of the BBB [74]. NPs were labelled with FITC to allow their tracking, and confocal images showed that the internalization of P80 coated NPs was significantly higher than uncoated NPs, suggesting higher uptake due to the P80 coating [16]. However, the presence of positive charges on the surface of P80-coated NPs may affect their in vivo biodistribution, so further studies are needed to assess the efficiency of this vehicle to transport Mtx to the brain.

Nagpal et al. [75] employed P80-coated CsNPs for the transport of Rivastigmine to the brain. This drug is a reversible cholinesterase inhibitor that improves memory in Alzheimer’s disease but is unable to cross the BBB [76]. A two-factor, three-level (32) central composite design was used in order to optimize the size, ZP and drug loading efficiency of NPs. The resulting NPs had a size of 154 nm, ZP of 26 mV and drug loading efficiency of 96%. The coating with P80 did not have any effect on the physico-chemical properties of NPs, even though P80 was added to the solution of NPs, similarly to the method adopted by Soni et al. [73] and Azadi et al. [77]. In vivo experiments were performed on mice and the effect of Rivastigmine-loaded P80-coated CsNPs on their memory was evaluated. After seven days administration, the mice showed enhanced memory activity due to a decreased brain acetyl-cholinesterase activity when compared to mice treated with Rivastigmine loaded into uncoated NPs or free Rivastigmine. This showed that the coating with P80 was advantageous for the management of Alzheimer’s disease [75].

Azadi et al. [77] aimed to deliver Mtx loaded in P80-coated CsNPs to treat CNS malignancies [78]. Mtx is an anti-cancer agent with poor permeability across the BBB, since it is a substrate of efflux transporters [79]. CsNPs were generated by ionic gelation and coated with 1% (*v*/*v*) P80, resulting in a lower ZP (+16 mV) and size (106 nm) as compared to uncoated NPs (ZP +21 mV and size 118 nm). Results in this study suggest that the coating with P80 may help to decrease the electrostatic interactions between chitosan chains by masking some positive charges. A neuropharmacokinetic investigation was then carried out to discover the brain distribution of Mtx [77]. The formulations were i.v. administered to rats whose brains, livers, and plasma were analysed for drug concentration. Results showed that high Mtx concentrations in the brain were due to a high concentration of Mtx in the plasma, suggesting the low clearance of NPs by the RES. This was confirmed by the low concentration of Mtx in the liver, being an organ of the RES. The concentration of Mtx was high for both P80-coated and uncoated CsNPs as compared to free Mtx. However, P80-coated CsNPs produced a significantly higher brain uptake than uncoated CsNPs, indicating that P80 coating enhanced the penetration of Mtx through the brain [77].

More recently, Ray et al. [80] used P80-coated CsNPs to deliver ropinirole hydrochloride (RH) for the treatment of Parkinson’s disease, enhancing RH brain uptake [81]. Indeed, crosslinking RH with CsNPs is needed, since RH has a very short bioavailability and half-life after oral administration and does not cross the BBB after parenteral administration [82]. For the coating process, RH loaded NPs were resuspended in the phosphate buffer saline (PBS, pH 7.4) containing 1% *w/v* solution of P80. The resulting NPs were slightly larger (240 nm) than uncoated ones (233 nm), which indicated that the coating with P80 was successful. In vivo biodistribution studies in rats were performed and the concentration of RH was higher in the brains of mice that were treated with RH loaded into P80-coated CsNPs than uncoated CsNPs. Further, the brain/blood ratio of RH loaded in P80-coated CsNPs was higher than for RH loaded into uncoated CsNPs. All of these findings indicate that P80-coated CsNPs were able to target the brain by crossing the BBB owing to the coating with P80 [80].

However, the exact mechanisms of drug delivery to the brain by P80-coated CsNPs have not been elucidated in any of the studies above. Therefore, questions on whether P80-coated CsNPs are either engulfed by RMT or by blocking the efflux transporters remain to be answered. Finally, from the studies above, it seems that P80 acts as a key player in the delivery of drugs through the BBB. Hence the role that chitosan’s properties, such as mucoadhesion, play in this nano system is not well understood, especially in the case where the surface of the NP is entirely coated with P80. Further studies comparing NPs that are based on chitosan with others biopolymers, such as polylysine, following coating with P80 would be beneficial.

## 6. Other Chitosan-Based Nanocarriers

### 6.1. Microemulsions (ME)

**Figure 6:**
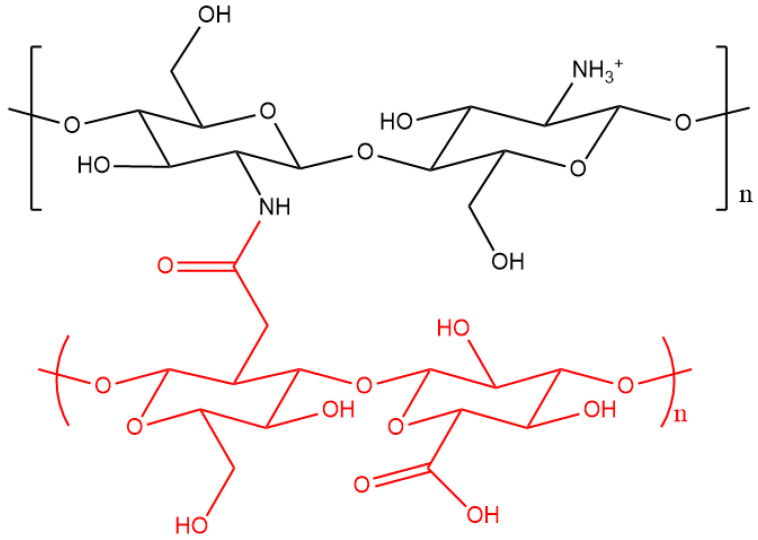
Structure of chitosan functionalised with hyaluronic acid (shown in red).

MEs are dispersions that consist of two immiscible liquid phases such as oils and water, mixed by mechanical means and using surfactants, so as to form a thermodynamically stable isotropic system with diameters that range from 10 to 50 nm [83]. Yao et al. [84] employed two soluble chitosan derivatives, chitosan hyaluronate (HaCs) (Figure 6) and chitosan hydrochloride (HcCs), in order to generate MEs loaded with nobiletin as a potential brain-selective delivery system. The resulting HaCs MEs had a diameter of 11 nm and negative ZP, while HcCs MEs had a diameter of 12 nm and positive ZP. In vivo biodistribution studies were carried out by i.v. injection of nobiletin (in the free form or loaded in MEs) to mice. Nobiletin is an anti-inflammatory drug, potentially useful to treat brain tumours. However, its application is restricted by the fact that it acts as a substrate for the efflux transporters on the BBB [85]. The concentrations of nobiletin in the plasma and main organs (brain, spleen, liver, kidney, and heart) were measured, and compared to the control groups; mice that were treated with HaCs MEs yielded the highest values of nobiletin in the plasma and brain, whilst the lowest ones were in the RES organs. These results suggested that HaCs MEs could increase the concentration of nobiletin in the plasma and, hence, also in the brain, since they were able to permeate the BBB, as shown by Yao et al. [86]. Indeed, HaCs MEs showed a prolonged retention time in the blood, owing to their anionic surface when compared to the cationic HcCs MEs. This suggests that the functionalisation of chitosan with hyaluronic acid plays a key role in reducing the uptake of micelles by the RES, in turn increasing their accumulation in the brain. Indeed, HaCs has been shown to reduce the absorption of serum proteins upon in vivo conditions; hence, it could be an alternative to the polymerization of chitosan with PEG [87]. However, under in vivo conditions, anionic NPs can adsorb cationic proteins in the blood, such as albumin, which may limit the cellular uptake of the drug delivery system; hence, further studies are needed.

### 6.2. Micelles

Pluronic micelles are made of ethylene oxide-propylene oxide block copolymers and they have received significant attention in the last few decades for application in oncology. These materials are able to self-assemble forming nano-sized core-shell aggregates, solubilizing hydrophobic drugs within their core [88]. Efficient brain tumour targeting can be achieved by conjugating chitosan on the micelles’ surface along with a brain ligand, in order to allow for AMT and RMT to occur [89,90]. For instance, Kim et al. [91] generated pluronic micelles for brain targeting modified with chitosan and a rabies virus glycoprotein (RVG), which is a short peptide binding to the acetylcholine receptor on neuronal cells [92]. NCs were labelled with Cy5.5 to monitor their fate following i.v. injection to mice. Fluorescence images of mice that were treated with pluronic micelles modified with chitosan and RVG showed a stronger signal in their head than the controls, indicating a need for the presence of both chitosan and RVG to efficiently target the brain. The NCs’ size apparently played a key role, since the obtained micelles were in the range of 60 nm [91]. The ability of these micelles to transport a protein, the enzyme β-galactosidase, and its accumulation in the brain was assessed following i.v. administration to mice. There was stronger and more prolonged histochemical detection of the enzyme activity in the brain, after loading the enzyme into pluronic micelles that were modified with chitosan and RVG, compared to the control. Overall, the results indicate that these micelles were suitable for protein drugs delivery across the BBB to the brain [91].

A study that was performed by Wang et al. [90] developed pluronic micelles that were modified with chitosan and loaded with myricetin for the treatment of glioblastoma multiforme (GMB). Micelles with an average size of 60 nm and a ZP of 30 mV were generated. Their cellular uptake in an in vitro model of the BBB showed the better transport of myricetin loaded in micelles than for free myricetin (control). The brain uptake of myricetin-loaded or unloaded micelles was also investigated following intragastric administration to mice. Again, enhanced brain penetration was achieved for myricetin that was loaded into the micelles when compared to free myricetin, confirming the ability of such micelles to enhance drug delivery across the BBB. The authors [90] suggested that the brain’s increased drug uptake after loading in micelles was due to their ability to inhibit the P-gp. However, in this study there is a lack of data showing the behaviour of myricetin loaded in micelles that were not modified with chitosan. Therefore, the role played by chitosan is not clear in the context of pluronic micelles which have been described as promising candidates for drug delivery to the brain on their own [93].

**Figure 7:**
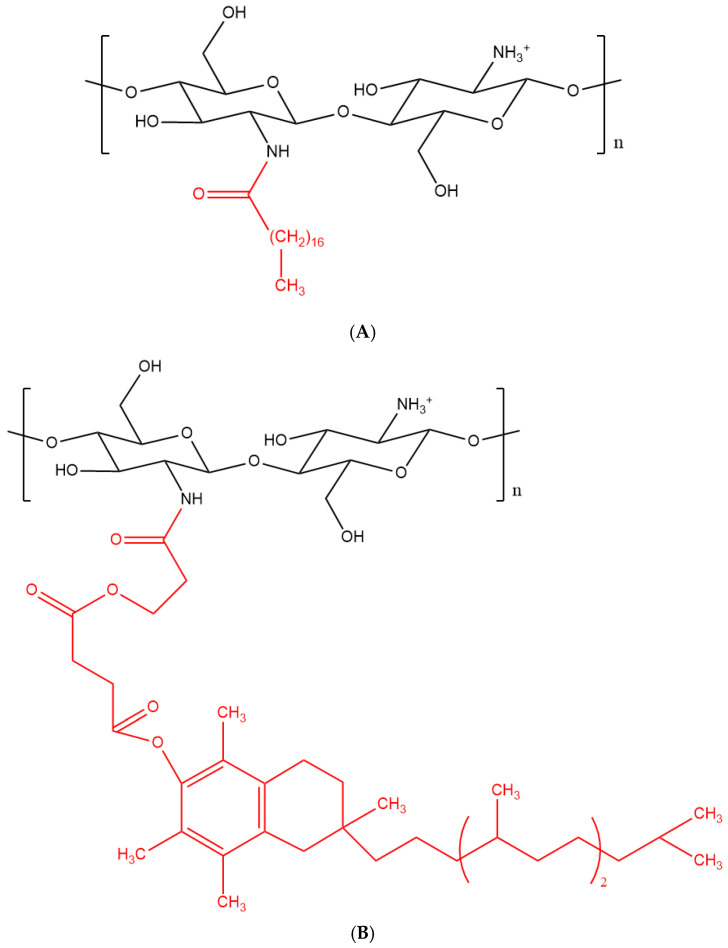
Structure of chitosan functionalised with (**A**) stearic acid (shown in red) and (**B**) d-α-tocopherol PEG 1000 succinate (shown in red).

Xie et al. developed another brain delivery system that was based on chitosan micelles [94]. Stearic acid (Sa) was conjugated to the chitosan backbone (Sa-Cs) (Figure 7A) to allow the formation of micelles [95]. The micelles were then labelled with FITC to enable their visualization during cellular uptake studies, employing an in vitro murine BBB model. There was a gradual and significant increase in fluorescence in the cell monolayer upon treatment with Sa-Cs micelles, suggesting their ability to cross the endothelium through endocytosis. In vivo imaging was also performed following i.v. administration of Sa-Cs micelles (labelled with the fluorescent dye DiR) to mice. Fluorescence microscopy showed that the micelles accumulated in the brain as soon as 5 min. after injection, revealing their strong ability to cross the BBB. Biodistribution studies were performed following i.v. administration of micelles that were loaded with Doxorubicin (Dox) to rats, and the drug was found to distribute quickly throughout the brain. Collectively, these results indicated that Sa-Cs micelles were able to cross the BBB both in vitro and in vivo and could be employed to deliver drugs to the brain [94]. However, the Dox level in the brain decreased 15 min. after injection while increasing in the lung, liver, and spleen, probably due to the increased uptake of macrophages of the RES. This result needs further investigation.

For brain-targeted cancer treatment, Agrawal et al. [96] developed chitosan micelles that were conjugated with transferrin and d-α-tocopherol PEG 1000 succinate (Figure 7B). This showed several benefits, including increased solubility of chitosan in acidic environments, the enhanced permeation of the NCs due to reversible opening of TJs, and inhibition of the P-gp [97,98,99,100]. The resulting micelles had an average size of 15 nm and an average ZP of −3 mV. Docetaxel was loaded as an anticancer model drug and coumarin-6 was encapsulated in the micelles to allow for their visualization during cell uptake studies. Glioma cells were employed in order to assess the cellular uptake of the micelles, and stronger fluorescence was recorded for those micelles than for the control (free coumarin-6 and chitosan micelles with no transferrin). Moreover, the micelles’ pharmacokinetics was evaluated following i.v. administration to rats. There was an increased plasma circulation time for such micelles when compared to the control, suggesting their potentiality to extravasate from the blood capillaries [96]. However, no in vivo brain uptake studies were undertaken in this investigation, since it had previously been reported that polymeric micelles smaller than 20 nm were suitable for brain targeted cancer therapy [96]. Finally, a biodistribution investigation can further help to understand the impact that the negatively charged micelles have on their retention time in the blood under in vivo conditions.

## 7. Conclusions

The delivery of therapeutic agents for the treatment of brain tumours or other disorders is still a challenge due to the presence of the BBB, which prevents most of the drugs from reaching the brain. CsNCs are promising candidates for the transport of agents to the brain due to their ability to cross the BBB by AMT, owing to chitosan’s mucoadhesive properties that were conferred by the free positive amino groups. The functionalisation of CsNPs with targeting molecules expressed on the BBB, such as Tf-R or apoliprotein receptors, has also been shown to increase the passage of NCs through the BBB. Moreover, the addition of methyl groups or alkylglyceryl groups to the chitosan backbone enhanced the ability of NCs to deliver drugs to the brain by increasing the mucoadhesion and hydrophobicity of the NC. Chitosan-based micelles or microemulsions were also found to be promising brain-targeting delivery systems due to their high brain biodistribution in in vivo studies and their ability to cross the BBB. However, CsNPs are known to be very unstable in the blood circulation, since the positive charges on chitosan backbone induce the adsorption of serum proteins in vivo, activating the macrophages of the RES. Only two studies, here described, have considered the possibility that the formulated drug delivery system can activate the RES and, indeed, PEGylation has actually been performed [35,60]. However, it is well known that one of the disadvantages of PEGylation is the formation of anti-PEG antibodies, which can activate the complement cascade and reduce the drug therapeutic efficiency [101]. Micelles that are coated with hyaluronic acid-conjugated chitosan can reduce the activation of the RES, since this chitosan derivative was shown to reduce the adsorption of serum proteins on the NP’s surface and to be a safe alternative to PEG [87].

In conclusion, it has been shown that the functionalisation of CsNCs plays a key role in enhancing their transport through the BBB for the delivery of drugs to the brain. However, further studies are needed in order to establish their suitability as drug delivery system in clinical applications.
